# Siderophore-Mediated Iron Acquisition Plays a Critical Role in Biofilm Formation and Survival of *Staphylococcus epidermidis* Within the Host

**DOI:** 10.3389/fmed.2021.799227

**Published:** 2021-12-24

**Authors:** Fernando Oliveira, Tânia Lima, Alexandra Correia, Ana Margarida Silva, Cristina Soares, Simone Morais, Samira Weißelberg, Manuel Vilanova, Holger Rohde, Nuno Cerca

**Affiliations:** ^1^LIBRO - Laboratory of Research in Biofilms Rosário Oliveira, Centre of Biological Engineering, University of Minho, Braga, Portugal; ^2^Institut für Medizinische Mikrobiologie, Virologie und Hygiene, Universitätsklinikum Hamburg-Eppendorf, Hamburg, Germany; ^3^i3S – Instituto de Investigação e Inovação em Saúde, Universidade do Porto, Porto, Portugal; ^4^REQUIMTE-LAQV, Instituto Superior de Engenharia do Porto, Instituto Politécnico do Porto, Porto, Portugal; ^5^IBMC, Instituto de Biologia Molecular e Celular, Universidade do Porto, Porto, Portugal; ^6^ICBAS-UP, Instituto de Ciências Biomédicas de Abel Salazar, Universidade do Porto, Porto, Portugal

**Keywords:** biofilms, virulence, iron, siderophores, mutagenesis, *Staphylococcus epidermidis*

## Abstract

Iron acquisition through siderophores, a class of small, potent iron-chelating organic molecules, is a widely spread strategy among pathogens to survive in the iron-restricted environment found in the host. Although these molecules have been implicated in the pathogenesis of several species, there is currently no comprehensive study addressing siderophore production in *Staphylococcus epidermidis*. *Staphylococcus epidermidis* is an innocuous skin commensal bacterium. The species, though, has emerged as a leading cause of implant-associated infections, significantly supported by an inherent ability to form biofilms. The process of adaptation from skin niche environments to the hostile conditions during invasion is yet not fully understood. Herein, we addressed the possible role of siderophore production in *S. epidermidis* virulence. We first identified and deleted a siderophore homolog locus, *sfaABCD*, and provided evidence for its involvement in iron acquisition. Our findings further suggested the involvement of siderophores in the protection against oxidative stress-induced damage and demonstrated the *in vivo* relevance of a siderophore-mediated iron acquisition during *S. epidermidis* infections. Conclusively, this study addressed, for the first time in this species, the underlying mechanisms of siderophore production, highlighting the importance of a siderophore-mediated iron acquisition under host relevant conditions and, most importantly, its contribution to survival within the host.

## Introduction

Iron, one of the most abundant metals on Earth, is a key nutrient for almost all living organisms, including bacteria (1). In the human host, iron is not readily accessible to invading pathogens, either because it forms insoluble ferric hydroxide complexes under aerobic neutral pH conditions or because it is mostly bound to host-derived proteins (e.g., transferrin and ferritin) (2, 3). Collectively, this represents an effective host defense mechanism against microbial infections, which is commonly referred to as nutritional immunity (4). With very few exceptions (5, 6) most bacteria rely on their ability to scavenge iron in order to survive in environments where the availability of this metal is limited. A common bacterial acquisition strategy is the production of siderophores, a class of small (usually <1 kDa), potent iron-chelating molecules with high affinity for Fe^3+^ (7). Siderophores comprise an impressive diversity of molecules and can be classified according to the functional groups constituting the iron-coordinating ligands as catecholates, hydroxamates, carboxylates, and mixed ligands (8). After being synthesized intracellularly and secreted into the environment as iron-free compounds, siderophores can compete for this element against host iron-binding proteins owing to their high affinity for iron (9). Afterwards, siderophore-iron complexes are internalized by bacteria through dedicated uptake systems (10).

Siderophores have been implicated in the pathogenesis of major pathogens (11–13). A study by França et al. demonstrated that *Staphylococcus epidermidis* biofilm cells exhibit an increased transcription of genes putatively involved in iron acquisition when in contact with human blood, including *htsC* and *fhuC*, putatively part of an iron ABC transporter system, and a siderophore homolog locus, *sfaABCD* (14). This led to the hypothesis that iron may play a significant role during *S. epidermidis* infections (15). Surprisingly, little is known about the role of siderophores on the pathogenesis of *S. epidermidis*. Despite being considered a commensal organism that colonizes the human skin and mucosae, *S. epidermidis* has emerged as an important agent of nosocomial bloodstream infections, and these typically occur after implantation of medical devices (e.g., central venous catheters) (16). Colonization of artificial surfaces is promoted by the species' inherent ability to form biofilms, and indeed, biofilm formation is considered as the most prominent virulence trait in *S. epidermidis* infections (17). The treatment of biofilm related infections represents a significant challenge because bacteria within biofilms are significantly more tolerant to antimicrobial therapy and can also evade the host immune response (18, 19). Due to the lack of effective antibiofilm therapies, treatment usually requires removal of the device, causing a substantial increase in patient morbidity (20). In the present study, we sought to identify the genetic basis of siderophore biosynthesis in *S. epidermidis* and hypothesized that siderophore production is a key step accounting for the remarkable ability of this bacterium to survive and form biofilms in the iron-restricted environment found in the human host.

## Materials and Methods

### Strains, Plasmids, Antibiotics, and Culture Media

Bacterial strains and plasmids used in this study are described in Supplementary Table 1. Unless otherwise noted, strains were cultured at 37°C. For genetic manipulations, *Escherichia coli* was grown in Lysogeny Broth (LB: 10 g/L tryptone, 5 g/L yeast extract, 10 g/L NaCl). Staphylococci were grown in TSB (BD Diagnostic Systems, Heidelberg, Germany). Solid media were prepared by adding 1.5% (w/v) agar (BD) to the culture medium. For selection of plasmids and recombinant alleles, antibiotics (Sigma-Aldrich, St. Louis, MO, USA) were added to the medium at the following concentrations: ampicillin (100 μg/mL) for *E. coli* selection and plasmid maintenance; trimethoprim (30 μg/mL), spectinomycin (150 μg/mL) and erythromycin (10 μg/mL) for staphylococci selection; and chloramphenicol (10 μg/mL) for staphylococcal plasmids maintenance. Iron restriction was achieved by slightly modifying a chemically defined medium (CDM) recipe (21), in which its original iron source [ammonium iron (II) sulfate] was omitted. This culture medium is henceforth referred to as CDM(Fe–). Iron-enriched conditions were achieved either by using TSB or by supplementing CDM(Fe–) with 10 μM FeCl_3_ [CDM(Fe+)]. All solutions and media were made with water purified through a Milli-Q water purification system (Millipore, MA, USA).

### Genetic Manipulations and Construction of Mutant Strains

Standard DNA manipulations were performed essentially as described by Sambrook et al. (22). An allelic replacement strategy was used for the construction of four deletion mutants in *S. epidermidis* 1,457 strain. The list of primers used is shown in Supplementary Table 2 (full details available in Supplementary Information).

### Quantification of Bacterial Iron Content

Two-mL of cultures grown overnight in TSB (BD) were harvested by centrifugation at 5,000 × *g*, for 10 min at 4°C. Cells were washed twice in ultrapure water and diluted into CDM(Fe–) to an OD_640_ of 0.025 (~10^7^ CFU/mL) in disposable plastic tubes. Chloramphenicol was added to the growth medium of the plasmid-bearing strains for plasmid maintenance. Cultures were incubated at 37°C, 120 rpm (ES-20 Shaker-Incubator) for 24 h. Afterwards, cultures were harvested by centrifugation at 5,000 × *g*, for 10 min at 4°C and the pellet washed thrice with metal-free ultrapure water to remove salts. Samples were then assayed for total iron content through atomic absorption spectrophotometry (full details available in Supplementary Information).

### Detection of Siderophore Production

Bacterial cultures were prepared as described above, except that the incubation period in CDM(Fe–) was 72 h for induction of maximal siderophore production. Afterwards, cultures were harvested by centrifugation at 5,000 × *g*, for 10 min at 4°C. Culture supernatants were collected and filter-sterilized (pore size 0.2 μm) for analysis of siderophore production using a modified Chrome Azurol S (CAS) agar diffusion assay as previously described (23).

### Planktonic Growth Curves

Two-mL of cultures grown overnight in TSB were harvested by centrifugation at 5 000 × *g* for 10 min. Cells were washed twice in 0.9% (w/v) NaCl and diluted into CDM(Fe–) to an OD_640_ of 0.025 (~10^7^ CFU.mL^−1^) in a conical glass flask. Flasks were incubated at 37°C, 120 rpm (ES-20 Shaker-Incubator). OD_640_ was measured hourly up to 8 h and at 24 h of incubation [when appropriate, concentrated samples were diluted in CDM(Fe–) for accurate measurement]. Three independent experiments were performed for each condition tested.

### Biofilm Formation Assays

Biofilms were grown either on 96-well polystyrene microplates (Orange Scientific, Braine-l'Alleud, Belgium) for quantification of biofilm biomass, or on Lab-Tek^®^ Chamber Slide™System 8 Well Permanox^®^ Slides (Thermo Fisher Scientific Inc.) for confocal microscopy analysis. Cultures were prepared as described above, diluted into CDM(Fe–) to an OD_640_ of 0.25 (~10^8^ CFU/mL) and further diluted 1:100 into (i) CDM(Fe-); (ii) CDM(Fe+); or (iii) TSB. Afterwards, diluted bacterial suspensions were placed into the microplates/chamber slides and incubated for 24 h at 37°C under static conditions.

### Quantification of Biofilm Biomass

Biofilms formed on 96-well microtiter plates were used for biomass quantification. After incubation, culture supernatants were removed carefully, biofilms were washed twice with 200 μL of 0.9% NaCl, and then stained by crystal violet technique, as previously described (24). Experiments were run at least in triplicate with technical quadruplicates for each condition tested.

### Confocal Laser Scanning Microscopy Analysis

Biofilms formed on the chamber slide system were analyzed through confocal laser scanning microscopy (CLSM). After incubation, culture supernatants were removed carefully, biofilms were washed twice with 200 μL of 0.9% NaCl, and then stained with (i) DAPI nucleic acid stain (Sigma-Aldrich, MO, USA) for visualization of cells, and (ii) wheat germ agglutinin (WGA) conjugated with Texas Red (Thermo Fisher Scientific, Inc.) for staining of N-acetylglucosaminyl residues (PIA/PNAG). All staining procedures were performed according to the manufacturer's instructions. Stained biofilms were visualized as previously described (25).

### Gene Expression Analysis

RNA samples were obtained from *S. epidermidis* cells cultured in CDM(Fe-) for 24 h. RNA extraction, DNase treatment, and RNA quality determination were performed as previously described (25). cDNA synthesis was performed using the RevertAid First Strand cDNA Synthesis Kit (Thermo Fisher Scientific, Inc.) following manufacturer's instructions. The same amount of total RNA (300 ng) from each sample was reverse transcribed in a 10 μL reaction volume using random hexamer (or gene-specific) primers as priming strategy. To determine the possibility of genomic DNA carry-over, control reactions were performed under the same conditions but lacking the reverse transcriptase enzyme (NRT control). All RNA samples extracted were absent of significant genomic DNA. Gene expression was determined by quantitative real-time PCR (qPCR) using Xpert iFast SYBR Mastermix (GRiSP, Lda., Porto, Portugal) (full details available in Supplementary Information).

### Isolation of Peripheral Blood Mononuclear Cells

Human samples were obtained in agreement with the principles of the Declaration of Helsinki. PBMCs were isolated from human blood and used for monocyte purification by magnetic-activated cell sorting (full details available in Supplementary Information).

### Macrophage Differentiation

Monocytes (CD14^+^ cells) were plated in either 6-, 24- or 96-well cell culture plates (Nunclon™ Delta Surface, Thermo Fisher Scientific Inc.) in complete RPMI medium (cRPMI) (RPMI 1640 medium supplemented with 10 mM HEPES buffer, 2 mM L-glutamine, 100 U/mL penicillin/streptomycin, 0.05 mM β-mercaptoethanol (all from Sigma-Aldrich), 5% (v/v) heat-inactivated fetal bovine serum (FBS, Biowest, Riverside, MO, USA) or autologous plasma (where indicated). Cells were seeded at the appropriate concentration and incubated at 37°C in a humidified atmosphere and 5% CO_2_. To generate human monocyte-derived macrophages (hMDM) skewed toward an M1- or M2-like profile, cRPMI medium was supplemented with either 25 ng/mL of macrophage colony stimulating factor (M-CSF, R&D Systems, Minneapolis, MN, USA) or 25 ng/mL of granulocyte macrophage colony-stimulating factor (GM-CSF, PeproTech, Rocky Hill, NJ, USA), respectively. Half culture medium was replaced at days 3 and 6 of culture by fresh, prewarmed M-CSF or GM-CSF supplemented cRPMI medium, as adequate. Experiments using hMDM were performed with cells prepared from three different donors.

### Infection of Macrophages: Gentamicin Protection Assays

Suspensions of *S. epidermidis* wild-type (wt) and isogenic mutants were prepared as described above and used to infect previously plated RAW264.7 (5 × 10^5^ cells/well) or hMDM (1 × 10^5^ cells/well) in 96-well plates at a MOI of 10:1. To synchronize phagocytosis, plates were centrifuged at 300 × *g* for 2 min followed by incubation at 37°C in the presence of 5% CO_2_. Macrophages were allowed to internalize bacteria for 30 min. Afterwards, culture supernatants were discarded and pre-warmed serum-free cRPMI plus 50 μg/mL gentamicin (AppliChem, Darmstadt, Germany) was added for 60 min to eliminate extracellular bacteria. After this treatment, macrophages were rinsed with DPBS and further incubated in pre-warmed antibiotic-free cRPMI for the desired period of time (0, 2, 6, 12, and 24 h). Release of the gentamicin-protected bacteria (which corresponds to the intracellular fraction) was performed by lysing macrophages with 0.1% (w/v) saponin (Sigma-Aldrich) in PBS for 15 min. In order to eliminate bacterial aggregates, lysates underwent sonication (three cycles of 10 s at 30% amplitude using a Branson W140 Sonifier, Danbury, CT, USA). Lastly, lysates were serially diluted in PBS and plated onto TSA plates for CFU enumeration.

### Intracellular ROS Assay

Polymorphonuclear leukocytes (PMNs) were isolated from buffy coats from blood donations following a double gradient technique (full details available in Supplementary Information), and incubated (PMNs = 5 × 10^5^ cells) with *S. epidermidis* (5 × 10^6^ CFU/mL) for 15 or 60 min in 5 mL round-bottom tubes and stained with ROS-ID^®^ Total ROS/Superoxide detection kit (Enzo Life Sciences, Inc., Farmingdale, NY, USA) according to manufacturer's instructions. PMNs incubated with 50 nM phorbol 12-myristate 13-acetate (PMA, Sigma-Aldrich) were used as positive control for reactive oxygen species (ROS) production. Samples were analyzed in a BD FACSCANTO II flow cytometer, and data were analyzed using FlowJo software (v.10.5.3). Production of intracellular ROS was presented as the fold change in the mean fluorescence intensity (MFI) of PMNs challenged with the different mutants, compared to wt-challenged counterparts.

### Bacterial Survival After H_2_O_2_ Challenge

Bacterial suspensions were prepared as described above and adjusted in TSB to an OD_640_ of 0.025 (~1 × 10^7^ CFU/mL). Bacteria were incubated in TSB plus 0.5 mM H_2_O_2_ (Sigma-Aldrich) for 60 min at 37°C with shaking at 120 rpm. Bacterial aggregates were eliminated as described above, cells were serially diluted in PBS and plated on TSA plates for CFU enumeration. Data were obtained from three independent experiments.

### Catalase Activity

Catalase activity of bacterial cell lysates were determined has previously described by Videira et al. (26). The catalase activity was measured by following the decrease in absorbance at 240 nm for the consumption of H_2_O_2_ (ε240 nm = 43.6 M^−1^.cm^−1^) in a UV-3100 PC spectrophotometer (VWR, Portugal). Data were obtained from three independent experiments.

### Murine Model of *S. epidermidis* Bacteremia

Balb/c wild-type mice were purchased from Charles River (Barcelona, Spain) and kept under specific-pathogen-free conditions at the Animal Facility of the Instituto de Investigao e Inovao em Sade (i3s), Porto, Portugal. Procedures involving mice were performed according to the European Convention for the Protection of Vertebrate Animals used for Experimental and Other Scientific Purposes (ETS 123), directive 2010/63/EU of the European parliament and of the council of 22 September 2010 on the protection of the animals used for scientific purposes, and Portuguese rules (DL 113/2013). Experiments were approved by the institutional board responsible for animal welfare (ORBEA) at I3S and authorization to perform the experiments was issued by the competent national authority (Direco Geral de Alimentao e Veterinria) reference number 014036/2019-07-24. *In vivo* infection experiments were performed following biosafety level 2 (BSL-2) guidelines. BALB/c mice were infected intravenously with ~5 × 10^8^ CFU of *S. epidermidis* 1457 or its isogenic siderophore biosynthetic (Δ*sfa*) and iron uptake (Δ*hts* and Δ*fhu*C) mutants. Six hours after infection, mice were euthanized and blood, liver, kidneys, and spleen were aseptically collected, weighed, homogenized, and bacterial burden were determined by plating for CFU counts on TSA. Data was obtained from two independent experiments.

### Statistical Analysis

Statistical significance was determined using GraphPad Prism version 7.0a. The statistical tests used, significance values and group sizes are described in the figure legends. Significance was defined as *P* < 0.05, and data was only excluded on the basis of technical errors associated with the experiment.

## Results

### *Staphylococcus epidermidis* Has a Single Siderophore Biosynthetic Locus

**Figure 1:**
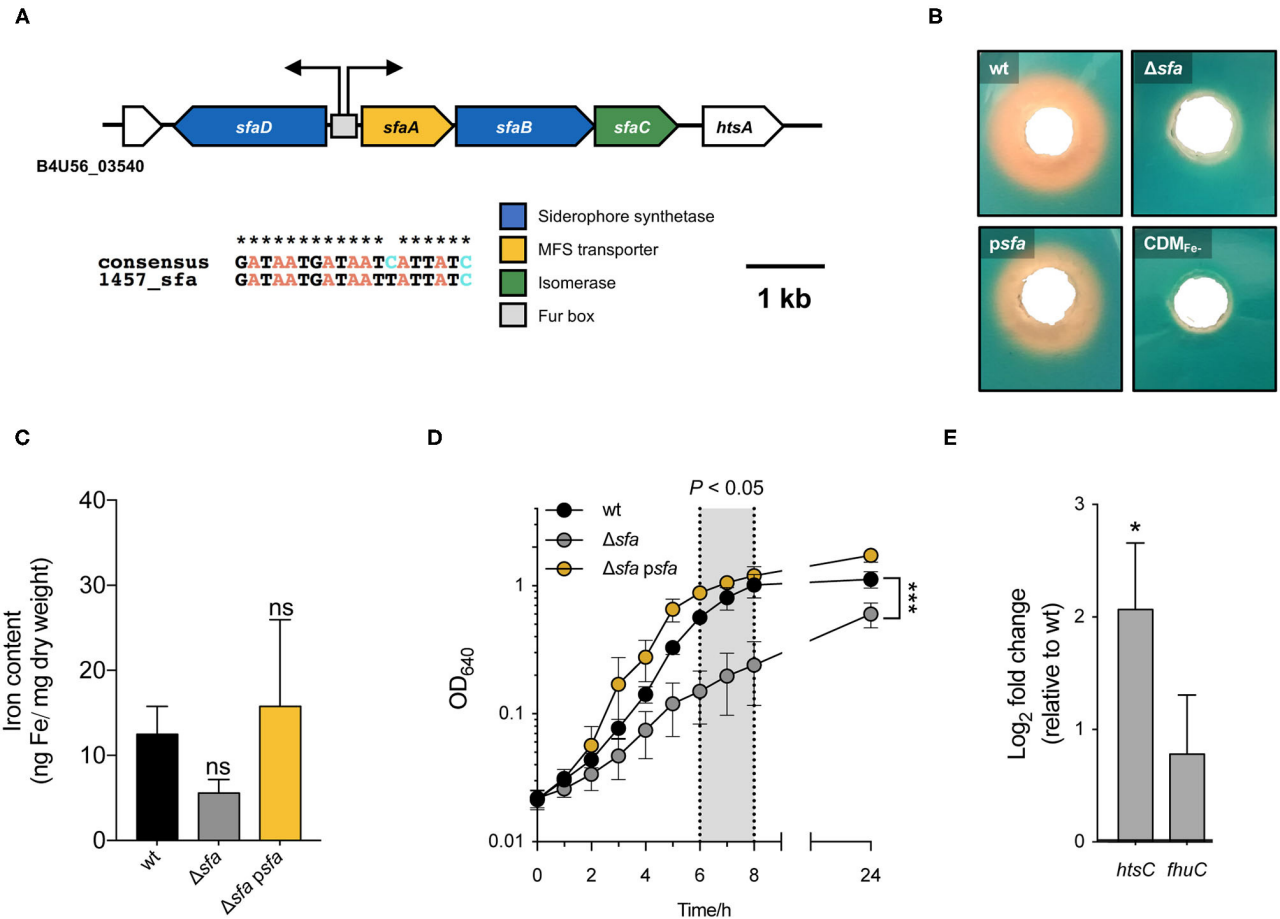
*S. epidermidis* has a single siderophore biosynthetic locus (*sfaABCD*) that plays an important role during growth under iron-restricted conditions. **(A)** Genomic organization of the sole siderophore biosynthetic locus in *S. epidermidis*. Assignments are based on the annotated genomes of *S. epidermidis* 1457 (accession number: CP020463). Open reading frames are indicated by arrows, which show the direction of transcription. Predicted transcriptional start sites are indicated by bent arrows. A putative Fur box is shown as a gray box and an alignment with a consensus sequence is provided below. The extent (in bp) of both deletion Δ*sfa* and DNA in complementing plasmid (p*sfa*) is shown above. **(B)** Wild-type (wt), deletion mutant Δ*sfa* and complemented strain p*sfa* were grown in CDM(Fe–) for 72 h at 37°C and the culture supernatants were tested for siderophore production using a modified CAS agar diffusion assay. The formation of an orange halo around each hole is indicative of siderophore presence in the supernatant. **(C)** Analysis of the cellular iron content by atomic absorption spectroscopy (*N* = 4). **(D)** Strains were allowed to grow for 24 h at 37°C, 120 rpm in CDM(Fe–) and growth was monitored as means of OD_640_. Shadowed area represents statistically significant differences between Δ*sfa* and wt strains (*N* = 3). **(E)** Transcription of iron acquisition-associated genes (*htsC* and *fhuC*) after culture in CDM(Fe-) for 24 h. Fold change data were calculated according to Pfaffl method and log-transformed (Log_2_). Values above and below 0 indicate up- and down-regulation of transcription, respectively, relative to wt (*N* = 3). *P < 0.05; ***P < 0.001; ns, not significant vs. wt. Two-tailed *t*-test **(C)** or two-way ANOVA **(D,E)**. The bars **(C,E)** and symbols **(D)** represent the mean of biological replicates and the error bars represent the s.e.m.

Analysis of *S. epidermidis* 1457 genome allowed the identification of a single putative siderophore biosynthetic gene cluster (B4U56_03545-03560). This gene cluster is ubiquitous in *S. epidermidis* [present in all genomes from a collection of *S. epidermidis* invasive and colonizing strains representing 31 STs (27)] and its genetic organization is conserved among different strains (Supplementary Figure 1). It was previously reported that the transcription of this cluster is iron-regulated (25). This observation, along with results obtained in *S. aureus* (28), led us to hypothesize that this gene cluster is involved in siderophore biosynthesis in *S. epidermidis*. Based on sequence similarity and our experimental data, described below, this locus is henceforth referred to as *sfaABCD*. The locus comprises four genes encoding two different siderophore synthetases (*sfaB* and *sfaD*), an isomerase (*sfaC*) and a siderophore exporter (*sfaA*) (Figure 1A), and contains a 19-bp DNA sequence in the intergenic region between divergently oriented *sfaA* and *sfaD* genes with similarity to a Fur box consensus sequence (29). To experimentally confirm the involvement of this gene cluster in siderophore biosynthesis, a *sfaABCD* deletion mutant in *S. epidermidis* 1457 was constructed (6,058 bp deleted). We reasoned that an *S. epidermidis* mutant unable to synthesize any siderophore would exhibit absence of siderophore activity in the culture medium, a decreased iron cell content and, ultimately, reduced growth under iron-deficient conditions. To assess this, wt and Δ*sfa* strains were cultured under iron-deficient conditions {iron-restricted chemically defined medium [CDM(Fe–)]; iron content <15 nM, as determined by atomic absorption spectroscopy} to equivalent cell densities, and culture supernatants were assayed for the presence of siderophore activity (Figure 1B). Unlike the wt, the Δ*sfa* strain was unable to secrete any siderophore to the culture supernatant. Furthermore, deletion of *sfa* resulted in a lower iron cell content relative to wt, although the difference was not statistically significant (Figure 1C). The growth of this mutant in an iron-restricted medium was significantly compromised from 6 to 24 h of growth, although bacterial growth still occurred under these culturing conditions (Figure 1D). Siderophore production, iron cell content and growth phenotypes were restored through complementation *in trans* [p*sfa*; containing *sfaABCD* and its natural promoter (6,188 bp; Figures 1B–D)]. Of note, increased mRNA levels of genes that we found to be involved in iron acquisition (*htsC* and *fhuC*) were observed in the Δ*sfa* mutant (Figure 1E). This transcriptional response is likely an important compensatory mechanism for the lack of siderophore production, allowing the siderophore-deficient cells to obtain iron from the surrounding medium, even if in smaller amounts. Collectively, these findings highlight the pivotal role of siderophore biosynthesis for the maintenance of iron homeostasis and optimal growth in iron-restricted environments.

### Siderophore-Mediated Iron Acquisition Is Required for Biofilm Formation

**Figure 2:**
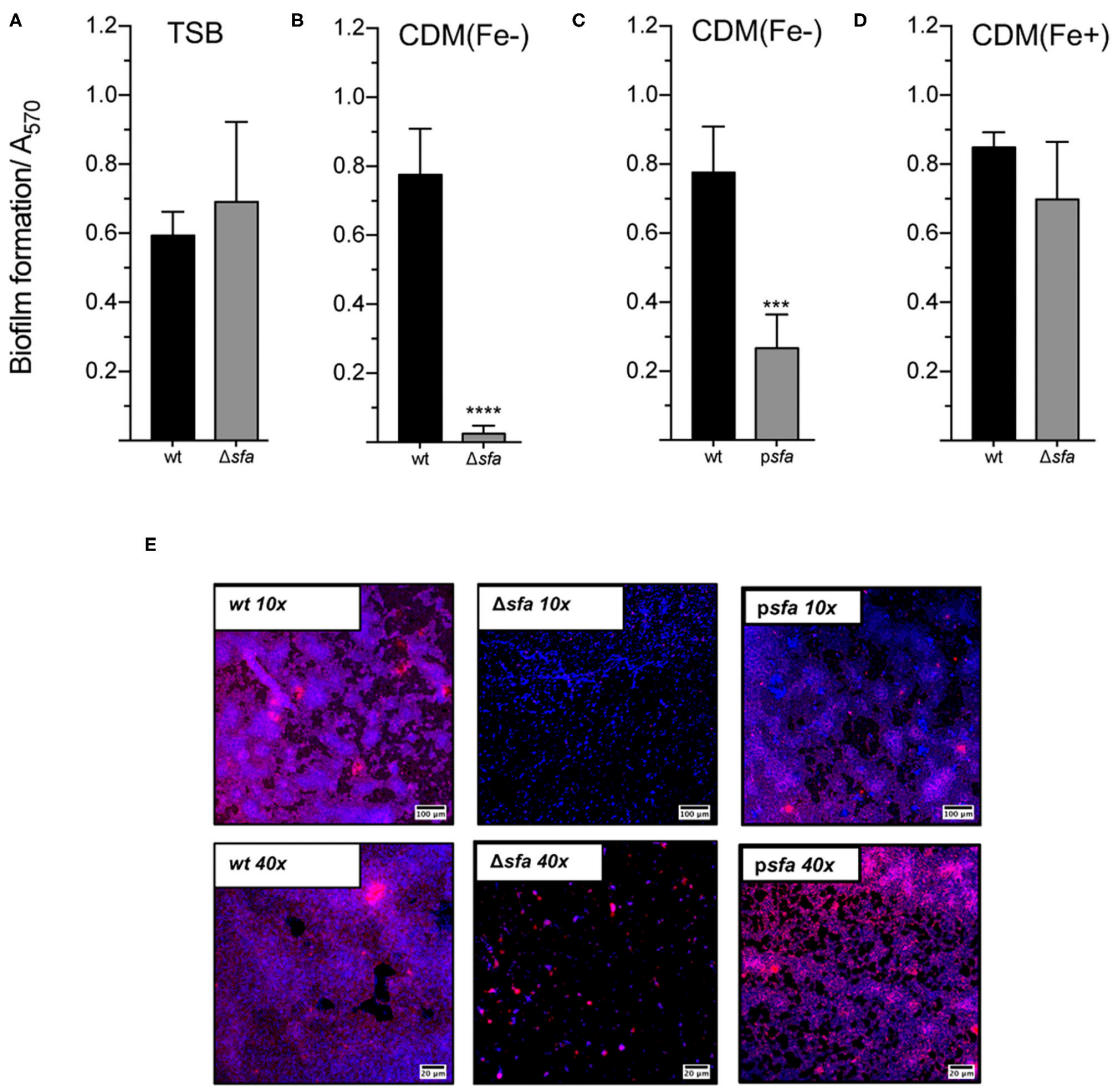
Siderophore biosynthesis is critical for biofilm formation under iron-restricted conditions. The biofilm formation ability of each strain was evaluated in **(A)**, TSB, **(B,C)**, iron-restricted chemically modified medium [CDM(Fe–)], and **(D)**, iron-enriched chemically modified medium [CDM(Fe+)]. Cells were allowed to grow statically for 24 h at 37°C on 96-well microplates. Biofilm quantification was performed through crystal violet staining (N = 3–5). **(E)** CLSM analysis of biofilms formed under iron-restricted conditions [CDM(Fe-)]. Biofilms were allowed to grow on an 8-well chamber slide system in CDM(Fe-) at 37°C for 24 h. CLSM was used for biofilm structure analysis and PIA/PNAG production after appropriate staining with DAPI (depicted in blue) and WGA-Texas Red (depicted in red). Representative images of Z-stack projections from two independent experiments are shown (scale bars = 100 μm for 10×; 20 μm for 40×). ***P < 0.001; ****P < 0.0001 vs. wt. One-way ANOVA with Dunnett's multiple comparisons test **(A–D)**. The bars represent the mean of biological replicates and the error bars represent the s.d.

We have previously demonstrated that iron availability plays a major role in *S. epidermidis* biofilms (25) and that PIA/PNAG production is almost abrogated under iron-restricted conditions (Supplementary Figure 2). In the present study, we explored the underlying mechanisms behind this iron dependence and tested the hypothesis that siderophores are required for biofilm formation by this pathogen. While the biofilm formation ability of the Δ*sfa* mutant was equivalent to that of the wt strain under iron-enriched conditions (Figure 2A), disrupted siderophore production proved to be detrimental for biofilm formation under iron-restricted conditions (Figure 2B), which are usually found in the human host (30). This detrimental effect could be partly to fully reversed either by providing the missing loci *in trans* (Figure 2C) or by supplementing an external iron source to the iron-restricted medium (Figure 2D). Biofilms formed under iron-restricted conditions were further examined through confocal microscopy for the assessment of biofilm organization and the PIA/PNAG content in the biofilm matrix (Figure 2E). While wt formed thick biofilms containing a high density of cells widespread across the surface and high amounts of PIA/PNAG, Δ*sfa* formed sparse biofilms mostly composed of cell clusters embedded in small amounts of PIA/PNAG. Gene complementation reversed the biofilm phenotype of the mutant strain. Taken together, our data reveals that *S. epidermidis* relies on a siderophore-mediated iron acquisition strategy to meet the increased iron demand imposed by the biofilm formation process in response to severe iron restriction.

### Siderophore Production May Confer Protection Against ROS-Induced Damage

**Figure 3:**
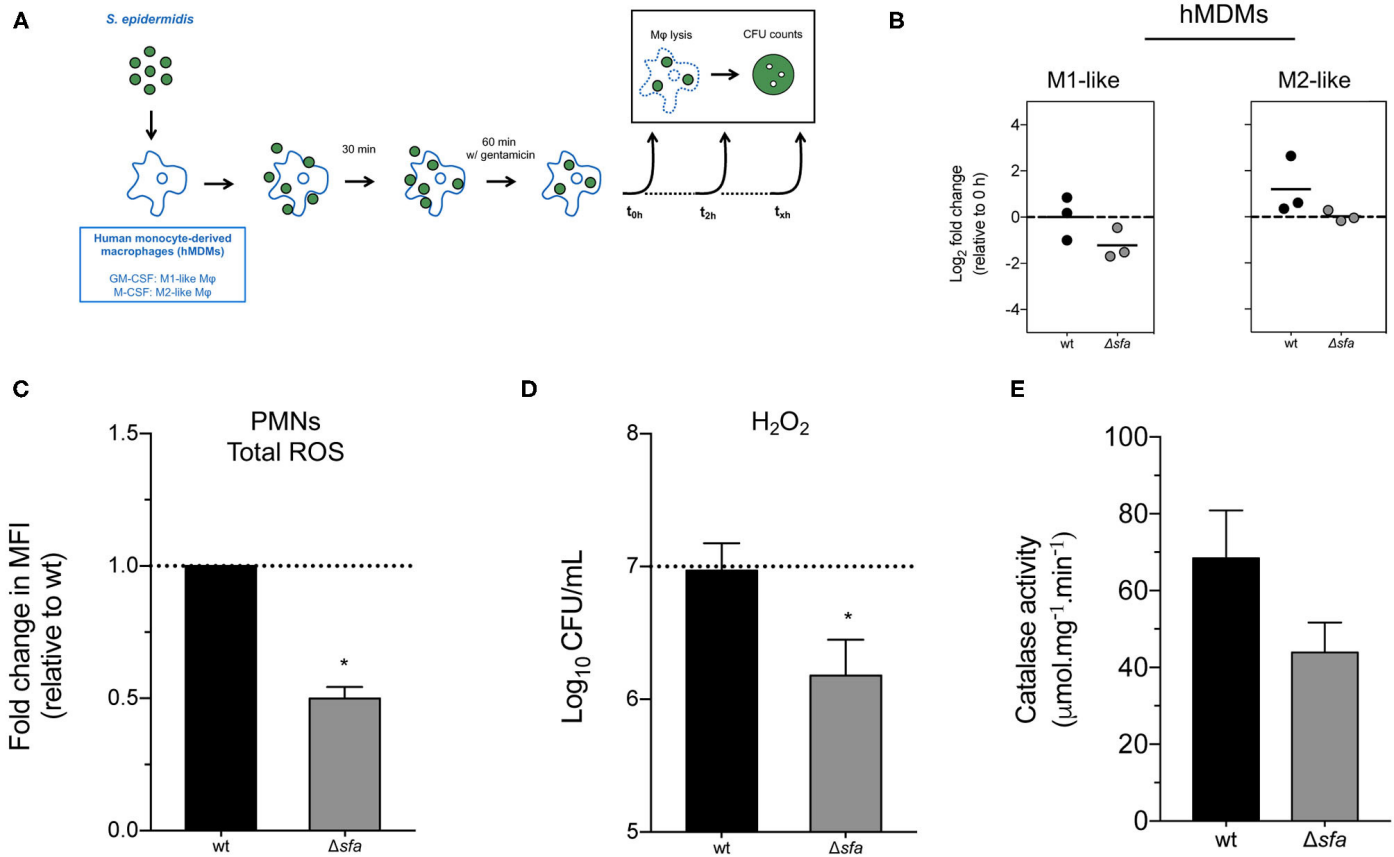
Contribution of siderophore production to the interaction between *S. epidermidis* and phagocytic cells. **(A)** Schematic diagram of the gentamicin protection assays used in this study. Human monocyte-derived macrophages (hMDMs) were infected with *S. epidermidis* as described in section Materials and Methods. Bacterial cells were recovered from the lysed macrophages, plated on TSA and colony forming units (CFU) counted after 1–2 days at 37°C. **(B)** Gentamicin protection assays using murine hMDMs. Each point represents the fold change in CFU (2 vs. 0 h post-infection) obtained in one experiment with a single donor and lines represent the median from biological replicates (*N* = 3 donors). Values above and below 0 indicate bacterial replication and clearance, respectively. No significant differences using one-way ANOVA with Dunnett's multiple comparisons test. **(C)** Human neutrophils were infected at a multiplicity of infection **(MOI)** of 10 and generation of total reactive oxygen species (ROS) was quantified after 60 min. Data is represented as mean ± SD (*N* = 2 donors) of the fold change in mean fluorescence intensity (MFI). **(D)** Susceptibility to H_2_O_2_-mediated killing. Dashed line represents starting bacterial concentration (1 × 10^7^ CFU/mL) (*N* = 4). **(E)** Catalase activity of bacterial cell lysates. *P < 0.05 vs. wt. The bars represent the mean of biological replicates and the error bars represent the s.d. **(C–E)**.

Macrophages and neutrophils are crucial players in the innate immune response against staphylococcal infections, as they phagocytose invading bacteria and expose them to a plethora of antimicrobial compounds (31). While biofilm formation has been shown to protect *S. epidermidis* from phagocytosis (32) and the action of antimicrobial peptides (33), the fate of *S. epidermidis* cells once phagocytosed is poorly understood. Our previous findings on the importance of iron availability for *S. epidermidis* growth and biofilm formation (25) led us to hypothesize that the lack of siderophore production may affect the survival of this bacterium inside phagocytic cells. To that end, we studied the ability of *S. epidermidis* to survive within human monocyte-derived macrophages (hMDMs) with different polarization status (M1- and M2-like macrophages) (Figure 3A). hMDMs cleared phagocytosed *S. epidermidis* by 24 h post-infection, as no CFUs were detected at this time point in both M1- and M2-like hMDM cultures (data not shown). Analysis of the number of bacterial cells residing intracellularly at 2 h post-infection revealed a numerical decrease of Δ*sfa* cells in M1-like macrophages (Figure 3B), although the difference was not statistically significant relative to wt. Additionally, we were equally interested in understanding how the single deletions we created in *S. epidermidis* would impact neutrophil function. Following infection (60-min challenge) of freshly isolated human neutrophils with *S. epidermidis* wt or its isogenic Δ*sfa* mutant, we detected a significant drop in ROS production by neutrophils co-incubated with Δ*sfa* (Figure 3C). Further experiments demonstrated the higher susceptibility of the Δ*sfa* mutant to hydrogen peroxide (H_2_O_2_) (Figure 3D), which could be partly explained by a lower catalase activity relative to wt (~35% reduction; (Figure 3E). Collectively, our findings suggest that siderophore production play a relevant role in the protection of *S. epidermidis* against ROS-induced damage.

### Siderophore-Mediated Iron Acquisition Contributes to the Survival of *S. epidermidis* Within the Host

**Figure 4:**
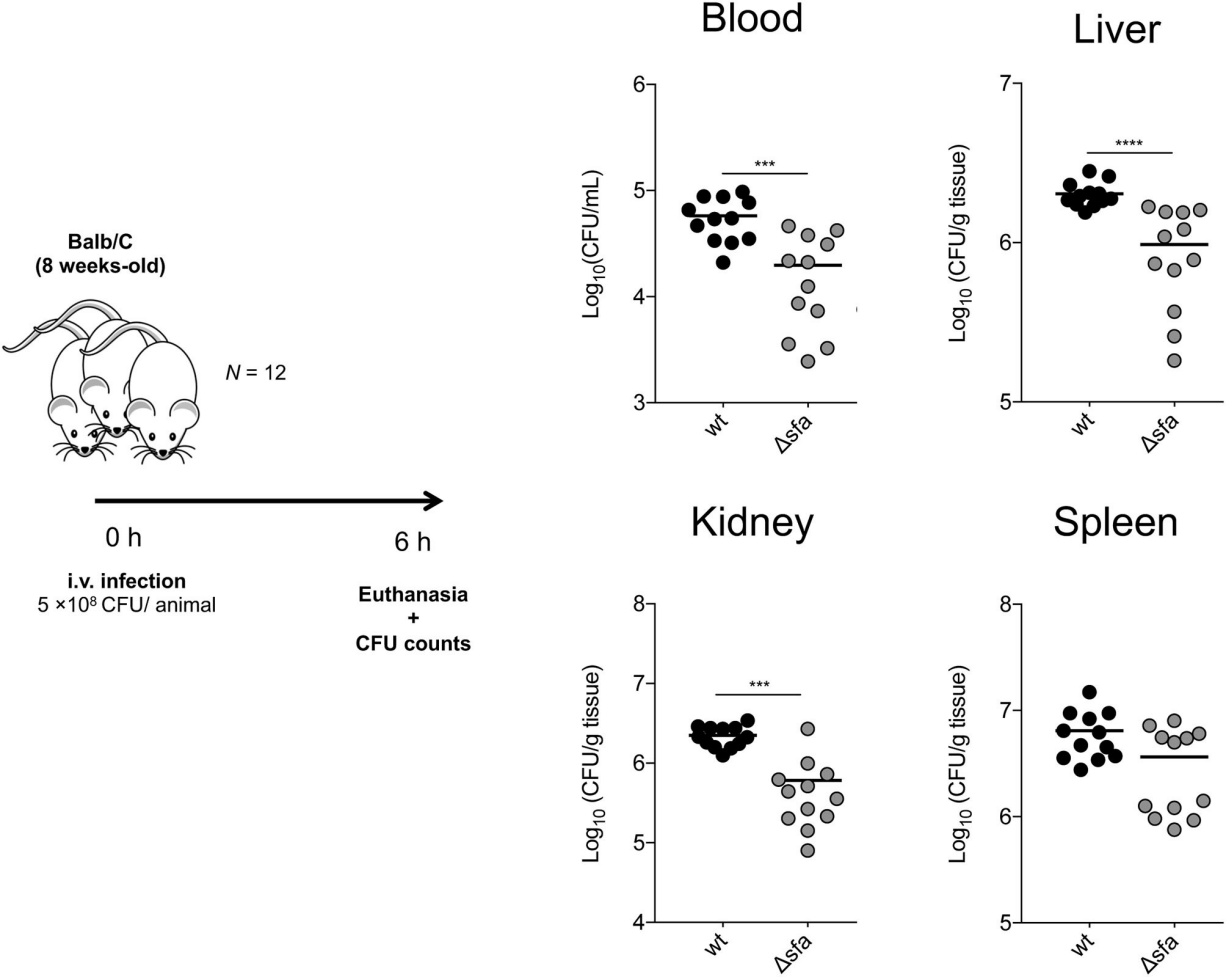
*S. epidermidis* deficient in siderophore biosynthesis display impaired survival within the host. BALB/c mice were infected intravenously with 5 × 10^8^ CFU of *S. epidermidis* 1457 or its isogenic siderophore biosynthetic mutant (Δ*sfa*). Six hours after infection, mice were euthanized and blood, liver, kidneys, and spleen were aseptically collected, weighed, homogenized, and bacterial burdens were determined by CFU counts. Each symbol represents one animal (*N* = 12/strain). Horizontal bars represent the mean from two pooled independent experiments. Significant differences were determined by one-way ANOVA with Dunnett's multiple comparisons test. ***P < 0.001, ****P < 0.0001 vs. wt. Data from the wt experiments was obtained during an experiment shared with (45).

Being a major cause of bloodstream infections originating from indwelling medical device contamination (34), we hypothesized that siderophore-mediated iron acquisition provides *S. epidermidis* with a competitive advantage within the host. By using a murine *in vivo* model of *S. epidermidis* bacteremia, we detected that mouse groups infected with the siderophore biosynthetic Δ*sfa* mutant exhibited significantly reduced bacterial loads not only in the bloodstream, but also in specific organs, such as liver and kidneys, 6 h post-infection, as compared with the wt strain (Figure 4). Conclusively, our *in vivo* findings provide significant evidence for the importance of siderophore-mediated iron acquisition in *S. epidermidis* survival within the host.

## Discussion

Although the role of iron in bacterial infections has long been established (3), iron acquisition in *S. epidermidis* is still massively underexplored. This comes as a surprise since this bacterial species is a major source of bloodstream infections (16), an environment where the bioavailability of iron is extremely restricted (30). In the present study we directed our attention toward siderophore production. Siderophore production have been reported long ago in staphylococci (staphyloferrins) (35), but the genetic information underlying siderophore biosynthesis was described only two decades later in *S. aureus* (28). Here we provide, for the first time in *S. epidermidis*, a clear link between the genetic loci *sfaABCD* and siderophore production. Available genomic information indicates that this locus is widespread across *S. epidermidis* strains and is likely the sole siderophore biosynthetic locus of this species. This is an important difference in comparison with *S. aureus*, which is known to produce at least two structurally different siderophores, staphyloferrins A and B (28, 36). Remarkably, although our Δ*sfa* mutant showed attenuated growth under iron-restricted conditions, it still presented a small amount of iron stored intracellularly that allowed replication. This observation supported the hypothesis that *S. epidermidis* expresses other iron uptake systems to meet its iron demand.

Conversely, we were able to demonstrate that the lack of siderophore production was absolutely detrimental for biofilm formation. The association between iron acquisition and biofilm formation has been established in some pathogens (15), and we have previously demonstrated the higher dependence of *S. epidermidis* on iron availability to form biofilms relative to planktonic growth (25). In this study, we underscored this association and show that siderophores play a functional role in *S. epidermidis* biofilm formation under iron-restricted conditions. This finding is of upmost importance, since biofilm formation is regarded as a major virulent trait in the context of *S. epidermidis* infections (37).

Interestingly, our observations also suggest that *S. epidermidis* siderophores may modulate ROS generation and participate in ROS detoxification. The observed higher H_2_O_2_ susceptibility in the absence of siderophore production is partly explained by a decreased catalase activity, although other, yet unknown mechanisms might be at play. It has been demonstrated, however, that by sequestering iron, siderophores reduces the iron availability in other reactions that lead to increased ROS production in several other bacterial pathogens (38–42). However, it is worth mentioning that these observations have been reported for chatecolate-type siderophores only, whereas similar findings in carboxylate-type siderophores, such as staphyloferrins, have not been found (43).

Lastly, by using an *in vivo* murine model of bacteremia, we demonstrated the key role of siderophore biosynthesis for *S. epidermidis* survival in the host. It was also interesting to note the absence of any significant organ-dependent variation in the bacterial loads of our siderophore-deficient mutant, suggesting that *S. epidermidis* siderophores do not play a niche-specific role, at least for the organs tested. This comes in contrast with recent observations in *S. aureus*, with siderophores being heterogeneously distributed across abscesses in different tissues (44). This not only underscores the differences between *S. epidermidis* and *S. aureus* regarding iron acquisition and siderophore production, but also it seems to imply that *S. epidermidis* siderophores play an equal role regardless of the host location.

Conclusively, this study represents the most thorough analysis to date of siderophore production by *S. epidermidis*, filling a gap of knowledge on iron acquisition in this pathogen. We provided evidence that *S. epidermidis* relies on a siderophore-mediated iron acquisition strategy to form biofilms and survive in the nutrient-deprived environment found in the host. Ultimately, this work emphasizes the potential of siderophore biosynthesis as a suitable target for the development of new therapeutic strategies against staphylococcal biofilm-associated infections.

## Data Availability Statement

The original contributions presented in the study are included in the article/Supplementary Material, further inquiries can be directed to the corresponding author.

## Ethics Statement

The studies involving human participants were reviewed and approved by Centro Hospitalar São João Ethics Committee. The patients/participants provided their written informed consent to participate in this study. The animal study was reviewed and approved by Direção Geral da Alimentação e Veterinária.

## Author Contributions

FO, MV, HR, and NC designed research and wrote the paper. FO, TL, AS, NC, and CS performed research. SM contributed new reagents/analytic tools. FO, TL, AC, CS, SM, and SW analyzed data. MV, HR, and NC supervised research. All authors read and approved the final version of the manuscript.

## Funding

This study was supported by the Portuguese Foundation for Science and Technology (FCT) through the funded project PTDC/BIAMOL/29553/2017, under the scope of COMPETE2020 (POCI-01-0145-FEDER-029553), and the strategic funding of UID/BIO/04469/2019 unit. This study was also supported through funds from the German Research Council (DFG) and the Damp Foundation. FO was supported by an individual Ph.D. scholarship (SFRH/BD/101399/2014).

## Conflict of Interest

The authors declare that the research was conducted in the absence of any commercial or financial relationships that could be construed as a potential conflict of interest.

## Publisher's Note

All claims expressed in this article are solely those of the authors and do not necessarily represent those of their affiliated organizations, or those of the publisher, the editors and the reviewers. Any product that may be evaluated in this article, or claim that may be made by its manufacturer, is not guaranteed or endorsed by the publisher.
