# Supplementary material for: Siderophore-Mediated Iron Acquisition Plays a Critical Role in Biofilm Formation and Survival of Staphylococcus epidermidis Within the Host
Source: Front Med (Lausanne). 2021 Dec 24;8:799227. doi: 10.3389/fmed.2021.799227 (PMC8738164; doi:10.3389/fmed.2021.799227)
Supplement: Supplementary file 1 [file Data_Sheet_1.docx]

Supplementary Material

**Siderophore-mediated iron acquisition plays a critical role in biofilm formation and survival of *Staphylococcus epidermidis* within the host**

**Fernando Oliveira^1,2,3^, Tânia Lima^3^, Alexandra Correia^3^, Ana Margarida Silva^3^, Cristina Soares^4^, Simone Morais^4^, Samira Weißelberg^2^, Manuel Vilanova^3,5,6^, Holger Rohde^2^, Nuno Cerca^1*^**

^1^Centre of Biological Engineering, LIBRO - Laboratory of Research in Biofilms Rosário Oliveira, University of Minho, Campus de Gualtar, 4710-057, Braga, Portugal

^2^Institut für Medizinische Mikrobiologie, Virologie und Hygiene, Universitätsklinikum Hamburg-Eppendorf, Martinistraße 52, 20246 Hamburg, Germany

^3^i3S - Instituto de Investigação e Inovação em Saúde, Universidade do Porto, Rua Alfredo Allen, 4200-135, Porto, Portugal

^4^REQUIMTE-LAQV, Instituto Superior de Engenharia do Porto, Instituto Politécnico do Porto, Rua Dr. António Bernardino de Almeida, 431, 4249-015 Porto, Portugal

^5^IBMC, Instituto de Biologia Molecular e Celular, Universidade do Porto, R. Alfredo Allen, 4200-135 Porto Porto, Portugal

^6^ICBAS-UP, Instituto de Ciências Biomédicas de Abel Salazar, Universidade do Porto, R. Jorge de Viterbo Ferreira 228, 4050-313, Porto, Portugal

# Supplementary Materials & Methods

## Sequence analysis

DNA sequences were retrieved from the available genome of *S. epidermidis* 1457 (NCBI accession no. CP020463). Identification of putative Fur boxes was performed with FIMO tool (1), using the default parameters and the 19 bp Fur box consensus sequence 5’ GATAATGATAATCATTATC 3’ (2) as the input motif. antiSMASH 5.1.0 was used for identification and analysis of secondary metabolite biosynthesis gene clusters (3). Synteny conservation was studied in all available *S. epidermidis* genomes using SyntTax (4).

## Genetic manipulations

Standard DNA manipulations were performed essentially as described by Sambrook *et al*. (5). Restriction endonucleases were purchased from New England Biolabs, Inc. (Frankfurt, Germany) or Thermo Scientific Inc (Waltham, MA, USA). Phusion High-Fidelity DNA Polymerase and DyNAzyme II DNA Polymerase were purchased from Thermo Scientific Inc. Plasmid DNA was purified using the QIAprep Spin Miniprep Kit (QIAGEN, Hilden, Germany) according to the manufacturer’s instructions. For plasmid purification from staphylococci, the resuspension buffer provided with the plasmid isolation kit was supplemented with 25 U of lysostaphin (Sigma-Aldrich), and the cell suspension was incubated for 30 min at 37°C. Oligonucleotides and DNA sequencing services were purchased from Eurofins Genomics (Ebersberg, Germany).

## Construction of mutant strains

An allelic replacement strategy was used for the construction of four deletion mutants in *S. epidermidis* 1457 strain. The list of primers used is shown in **Table S2**. For each mutant, two ~1kb fragments flanking regions up- and down-stream the coding region to be deleted and an antibiotic resistance cassette were amplified using Phusion High-Fidelity DNA Polymerase (Thermo Fisher Scientific Inc.). Amplicons were ligated and cloned into plasmid pBASE6 (18) using (i) Gibson Assembly^®^ Cloning Kit (New England Biolabs, Inc.), according to the manufacturer’s instructions, or (ii) circular polymerase extension cloning (CPEC), as previously described (6). Resulting plasmids were introduced by electroporation, first into *S. aureus* RN4220 and then into *S. epidermidis* 1457Δ*agr* or 1457-M12. Next, using phage A6C, plasmids were introduced into *S. epidermidis* 1457. Selection of mutants was performed essentially as described (7). Correctness of the chromosomal mutations was verified using PCR with primers that bind to genetic regions not involved in the mutagenesis process, and afterwards respective amplicons were sequenced. For complementation, DNA fragments containing the deleted coding sequences and their anticipated natural promoters were amplified and cloned into plasmid pRB473, as described above. Plasmids were introduced by electroporation first into S. aureus PS187ΔΔ and then into *S. epidermidis* 1457 mutant strains using phage Φ187, following a previously published protocol (8). All wild-type and mutated alleles were confirmed for their sequence correctness by DNA sequencing.

## Quantification of bacterial iron content

Homogenized bacterial samples dispersed in ultrapure water were placed in previously weighed Teflon vessels (MS105, Mettler Toledo, Switzerland), and then dried in an oven at 90ºC (P Selecta, Barcelona, Spain) until three reproducible weight values were obtained. Microwave-assisted digestion of samples was performed by adding 10 mL of Suprapur^®^ nitric acid 65% (v/v) (Merck) to each vessel containing the dried and accurately weighed samples. The microwave-assisted digestion proceeded accordingly with the steps described in **Table S3**, using a Mars-X 1500 W (Microwave Accelerated Reaction System for digestion and extraction, CEM Mathews, NC, USA), configured with a 14-position carousel and equipped with pressure and temperature sensors. After digestion, and cooling to approximately 30°C, samples were kept frozen in polycarbonate containers at -20°C until analysis. Lastly, Iron quantification was carried out using an Analytik Jena ContrAA 700 High-Resolution Continuum Source Flame Atomic Absorption Spectrometer (Analytik Jena, Jena, Germany) equipped with a xenon short-arc lamp XBO 301 (GLE, Berlin, Germany) with a nominal power of 300 W operating in a hot-spot mode as a continuum radiation source. Iron was analyzed at 248.3270 nm by using the Graphite Furnace module equipped with an MPE60 autosampler (Analytik Jena) and argon 5.0 purity grade (Linde, München, Germany) as the inert gas. Transversal and pyrolytically coated graphite tubes with integrated platforms were used. In order to obtain maximum absorbance and minimum background values, operational parameters were optimized and are presented in **Table S4**. External calibration curves were daily constructed based on, at least, six standard solutions of iron prepared from 1 000 mg/L stock solutions (Panreac Quimica SA, Barcelona, Spain). Magnesium nitrate hexahydrate (traceable to SRM from NIST; Merck) was used as a matrix modifier at 0.1% (w/v). All glassware and plastic material were soaked in nitric acid (50% v/v), thoroughly rinsed with ultrapure water and dried before use. The instrument performance was checked using analytical blanks and standards analyzed daily and regularly along with samples. Results were normalized to the cell dry weight.

## Gene expression analysis

Each PCR reaction contained 2 μL of 1:200 diluted cDNA or NRT control, 5 μL of master mix, 1 μL of primer mixture (in the final reaction, each primer was at 0.3 μM), and 2 μL of nuclease-free water. qPCR runs were performed on a CFX 96 (BioRad) with the following cycle parameters: 95 °C for 3 min, and 40 cycles of 95 °C for 5 s, and 60 °C for 25 s. Melt analysis was performed at the end to ensure the absence of unspecific products and primer dimer. All genes were quantified in duplicate for biological triplicates. The expression of the genes tested was normalized to the expression of the reference gene 16S rRNA. Data were log transformed (Log2) before statistical analysis was performed. Information about the primers used in this study is listed in **Table S2**. Primers were designed with the aid of Primer3 (9) using *S. epidermidis* 1457 genome sequence (NCBI accession no. CP020463.1) as template. mFold was used for prediction of secondary structures (10). No secondary structures were found for the operating temperatures used. Gene specificity of all primers was confirmed using Primer-BLAST (11). PCR amplification efficiency (E) for each gene has been previously determined (12).

## Isolation of peripheral blood mononuclear cells (PBMCs)

### Human samples were obtained in agreement with the principles of the Declaration of Helsinki. PBMC were isolated from surplus buffy coats, kindly provided by the Immunohemotherapy Department of Centro Hospitalar São João (CHSJ), Porto, Portugal. Procedures were approved by the Hospital Ethical Committee (Protocol 90/19). Informed written consent that the byproducts of their blood collections could be used for research purposes was obtained from the blood donors. Blood was diluted 1:2 in DPBS (Dulbecco’s Phosphate Buffered Saline, without calcium and magnesium, Sigma-Aldrich) and 6 mL were carefully layered onto 3-mL Histopaque-1077 (Sigma-Aldrich) and centrifuged (400g, 30 min, RT, break off) (Heraeus Megafuge® 1.0R, Heraeus, Hanau, Germany). The PBMC layer was carefully transferred into a clean conical centrifuge tube and washed with DPBS. The cell pellet was recovered in DPBS and cell concentration was determined.

## Monocyte purification by magnetic-activated cell sorting

Monocytes were purified from previously prepared PBMC suspensions using CD14 MicroBeads, human kit (Miltenyi Biotec, Bergisch Gladbach, Germany) and MS columns (Miltenyi Biotec) in a Mini MACS separator (Miltenyi Biotec), according to manufacturer’s instructions. Obtained CD14^+^ cells were counted using a hemocytometer.

## Isolation of polymorphonuclear leukocytes (PMNs)

### PMNs were isolated from buffy coats from blood donations (Banco de Sangue, Centro Hospitalar Universitário de São João, Porto, Portugal) following a double gradient technique. 3 mL of Histopaque-1077 (Sigma-Aldrich) were carefully layered over 3 mL of Histopaque-1119 (Sigma-Aldrich) in 15 mL conical centrifuge tubes. Afterwards, 6 mL of blood samples were carefully layered onto the upper Histopaque-1077 layer. Tubes were then centrifuged at 700g, for 30 min at RT, break off (Heraeus Megafuge® 1.0R, Heraeus, Hanau, Germany). After centrifugation, the following pattern was observed (from top to bottom): plasma, PBMCs, Histopaque-1077, PMNs, Histopaque-1119, and red blood cells. PMNs were carefully transferred into a clean conical centrifuge tube and washed with DPBS. Tubes were centrifuged (400g, 10 min, 4°C). Pelleted cells were resuspended in DPBS and counted in a hemocytometer.

# Supplementary Figures and Tables

## Supplementary Figures

| 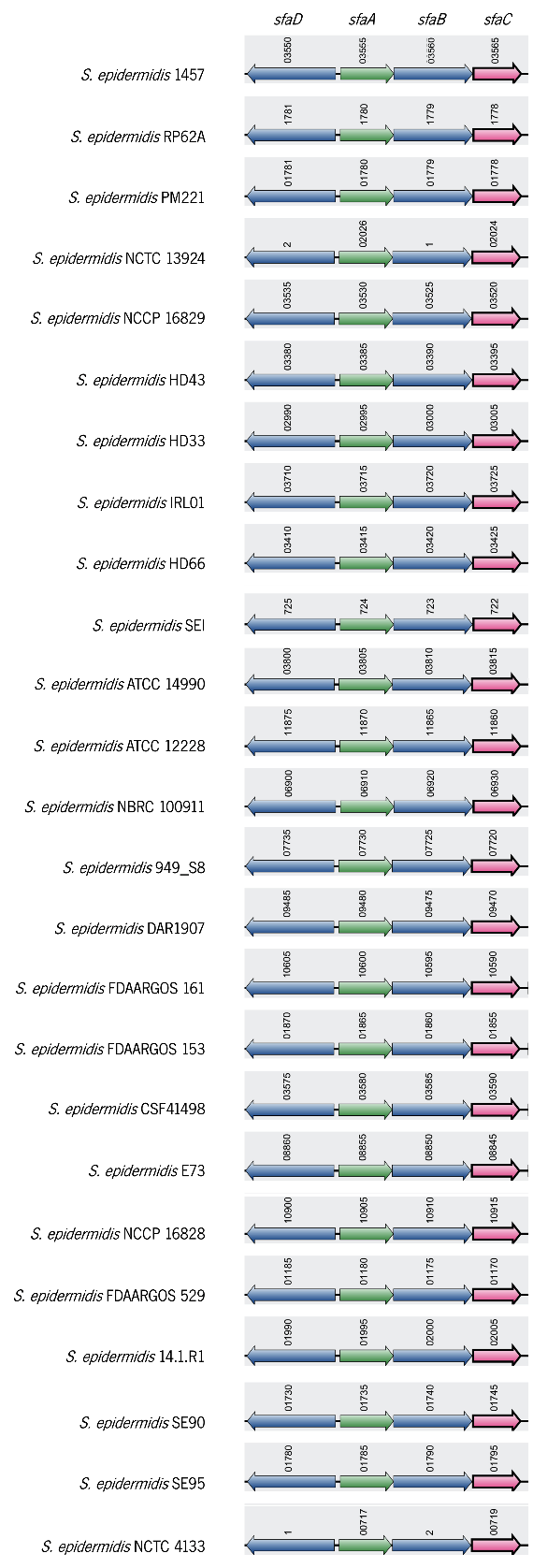 |
| --- |
| **Figure S1. Genomic organization of *sfa* locus in available *S. epidermidis* genomes.** Open reading frames are indicated by arrows, which show the direction of transcription. Genes encoding proteins of the same family are depicted in the same color. |
| 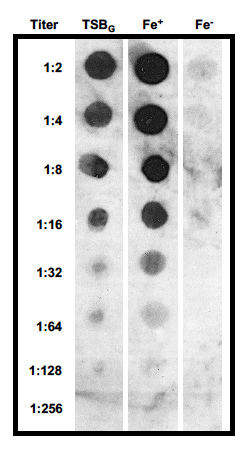 |
| **Figure S2. PIA/PNAG production is almost abrogated under iron-restricted conditions.** Quantification of PIA/PNAG production in *S. epidermidis* RP62A biofilms by dot blot analysis, as previously described (13). Serial dilutions of cell wall extracts were spotted onto PVDF membranes which were then incubated with WGA coupled to peroxidase. Bound WGA was then visualized by chemiluminescence. Fe-, iron deficiency; Fe+, iron excess. TSB_G_, TSB supplemented with 0.4% (w/v) glucose. |

## Supplementary Tables

| Table S1. Bacterial strains, plasmids and phages used in this study | | |
| --- | --- | --- |
| **Strain, plasmid or phage** | **Description*** | **Reference** |
| ***E. coli*** |  |  |
| DH5α | Chemically competent cells for cloning purposes | New England Biolabs (NEB) |
| TOP10 | Chemically competent cells for cloning purposes | Thermo Fisher Scientific, Inc |
| ***S. aureus*** |  |  |
| RN4220 | Derived from NCTC8325-4; r_k_^-^ m_k_^+^; accepts foreign DNA | (14) |
| PS187 Δ*hsdR* Δ*sauPSI* | *S. aureus* PS187 strain deficient in type IV and type I restriction systems | (15) |
| ***S. epidermidis*** |  |  |
| 1457 | Wyld-type clinical isolate from a central venous catheter infection; *icaADBC*^+^, *aap*^+^, *embp*^+^, strong biofilm formation | (16) |
| 1457Δ*agr*::*spcR* | Mutant carrying a deletion of the accessory gene regulator (*agr*) system; Spt^R^ | (17) |
| 1457Δ*sfa*::*spcR* | Mutant carrying a deletion of *sfaABCD;* Spt^R^ | This study |
| 1457Δ*sfa* p*sfa* | Complemented mutant 1457Δ*sfa*; *in trans* expression of *sfa* from its natural promoter; Spt^R^, Cm^R^ | This study |
|  |  |  |
| **Plasmids** |  |  |
| pBASE6 | Temperature-sensitive suicide mutagenesis vector; Amp^R^, Cm^R^ | (18) |
| pB-*sfa* | pBASE6 derivative containing *sfa*::*spcR*; Cm^R^, Spt^R^ | This study |
| pRB473 | Shuttle vector for cloning in *E. coli* and staphylococci; constitutive gene expression in staphylococci via *vegII* promoter | (19) |
| p*sfa* | pRB473 derivative containing *sfaABCD* and its natural promoter | This study |
|  |  |  |
| **Phages** |  |  |
| φ187 | *S. aureus* phage; wt | (20) |
| A6C | *S. epidermidis*; wt | (21) |
| **^*^**Abbreviations: Amp^R^, Cm^R^, Spt^R^, resistance to ampicillin, chloramphenicol, and spectinomycin, respectively. | | |

| Table S2. Sequences of oligonucleotides used in this study | | |
| --- | --- | --- |
| **Description** | **Purpose** | **Sequence (5’ - 3’)*** |
| **Δ*sfa*** |  |  |
| SERP1778_UP_Fw | Amplification of fragments for construction of plasmid pB-*sfa* | cactcatcgcagtgcagcgg**aattc**CTTTGTTTGTCATTATGAACATAC (*Eco*RI) |
| SERP1778_UP_Rv |  | tagagtcgacTAGTTCTTATTTACCTTTAACTCAAC |
| spcR_Fw |  | ataagaactaGTCGACTCTAGAGGATCGATC |
| spcR_Rv |  | ctttttgattGCATGCAAATGTCACTAATATTAATAAAC |
| SERP1781_DOWN_Fw |  | atttgcatgcAATCAAAAAGCACTTGAGC |
| SERP1781_DOWN_Rv |  | gcccgggtaccgagctccgg**aattc**GAGAGTATCCGTGCTGATATC (*Eco*RI) |
| SERP1776_Fw **¥** | Screening of plasmid integration through upstream region (5’) | GGAAGCACCTGCATTCACAC |
| pBASE6-spcR_Rv |  | ACTGTTCAATAAAGCTGACCGT |
| pBASE6_UP_INT_Fw | Screening of plasmid integration through upstream region (3’) | AGCTAGAGAGTCATTACCCCAG |
| SERP1778_Rv |  | GGCGAATGTTCGTGTCAAT |
| **p*sfa*** |  |  |
| SERP1781_Fw | Screening of plasmid integration through downstream region (5’) | TGGACCACTAGTGACGCAAA |
| pBASE6_DOWN_INT_Rv |  | CCTCGCAGCACGATATAAAG |
| spcR_2_Fw | Screening of plasmid integration through downstream region (3’) | AAGATGTCGCTGCAGAATGG |
| SERP1784_Rv **¥** |  | AAACCTACGCATCGCAAACC |
| SERP1778-81_Fw | Amplification of fragments for construction of plasmid p*sfa* | cccgccctgccactcatcgcagtgcagcgg**aattc**TTAGCACTGGGAATATATAGG (*Eco*RI) |
| SERP1778-81_Rv |  | actctagaggatccccgggtaccgagctcg**aattc**TCAATCTCTTGATGTATACCA (*Eco*RI) |
| **¥** These oligonucleotides were used for confirmation of locus deletion | | |
| ***** Restriction sites are underlined and in bold | | |

| **Table S3.** Microwave conditions for the digestion of bacterial samples | | | |
| --- | --- | --- | --- |
| **Ramp/ min** | **Pressure/ Psi** | **Temperature/ ºC** | **Hold/ min** |
| 5:00 | 150 | 50 | 10:00 |
| 10:00 | 200 | 100 | 10:00 |
| 10:00 | 200 | 140 | 15:00 |

| **Table S4.** Optimized operational parameters for the graphite furnace analysis of iron | | | | | |
| --- | --- | --- | --- | --- | --- |
| **Step** | **Name** | **Temperature/ ºC** | **Ramp/ ºC/s** | **Hold/ s** | **Time/ s** |
| 1 | Drying | 80 | 6 | 20 | 26.7 |
| 2 | Drying | 90 | 3 | 20 | 23.3 |
| 3 | Drying | 110 | 5 | 10 | 14.0 |
| 4 | Pyrolysis | 350 | 50 | 20 | 24.8 |
| 5 | Pyrolysis | 1100 | 300 | 10 | 12.5 |
| 6 | Gas adaptation | 1100 | 0 | 5 | 5.0 |
| 7 | Atomize | 2000 | 1500 | 4 | 4.6 |
| 8 | Clean | 2450 | 500 | 4 | 4.9 |

# References

1. Grant CE, Bailey TL, Noble WS. FIMO: Scanning for occurrences of a given motif. Bioinformatics. 2011;27(7):1017–8.

2. De Lorenzo V, Wee S, Herrero M, Neilands JB. Operator sequences of the aerobactin operon of plasmid ColV-K30 binding the ferric uptake regulation (*fur*) repressor. J Bacteriol. 1987;169(6):2624–30.

3. Blin K, Shaw S, Steinke K, Villebro R, Ziemert N, Lee SY, et al. antiSMASH 5.0: updates to the secondary metabolite genome mining pipeline. Nucleic Acids Res. 2019;

4. Oberto J. SyntTax: A web server linking synteny to prokaryotic taxonomy. BMC Bioinformatics. 2013;

5. J. Sambrook i D. W. Russell. Molecular cloning : a laboratory manual, III. Red. New York: Cold Spring Harbor Laboratory Press. 2001.

6. Quan J, Tian J. Circular polymerase extension cloning for high-throughput cloning of complex and combinatorial DNA libraries. Nat Protoc. 2011;6(2):242–51.

7. Bae T, Schneewind O. Allelic replacement in  *Staphylococcus aureus* with inducible counter-selection. Plasmid. 2006;55(1):58–63.

8. Winstel V, Kühner P, Rohde H, Peschel A. Genetic engineering of untransformable coagulase-negative staphylococcal pathogens. Nat Protoc. 2016;11(5):949–59.

9. Untergasser A, Cutcutache I, Koressaar T, Ye J, Faircloth BC, Remm M, et al. Primer3-new capabilities and interfaces. Nucleic Acids Res. 2012;40(15):e115.

10. Zuker M. Mfold web server for nucleic acid folding and hybridization prediction. Nucleic Acids Res. 2003;31(13):3406–15.

11. Ye J, Coulouris G, Zaretskaya I, Cutcutache I, Rozen S, Madden TL. Primer-BLAST: A tool to design target-specific primers for polymerase chain reaction. BMC Bioinformatics. 2012;13(1):134.

12. Oliveira F, França Â, Cerca N. *Staphylococcus epidermidis* is largely dependent on iron availability to form biofilms. Int J Med Microbiol. 2017;307(8):552–63.

13. Jefferson KK, Cerca N. Bacterial-bacterial cell interactions in biofilms: detection of polysaccharide intercellular adhesins by blotting and confocal microscopy. Methods Mol Biol [Internet]. 2006 [cited 2021 Nov 18];341:119–26. Available from: https://pubmed.ncbi.nlm.nih.gov/16799194/

14. Kreiswirth BN, Löfdahl S, Betley MJ, O’reilly M, Schlievert PM, Bergdoll MS, et al. The toxic shock syndrome exotoxin structural gene is not detectably transmitted by a prophage. Nature. 1983;305(5936):709–12.

15. Winstel V, Liang C, Sanchez-Carballo P, Steglich M, Munar M, Broker BM, et al. Wall teichoic acid structure governs horizontal gene transfer between major bacterial pathogens. Nat Commun. 2013;4:2345.

16. Mack D, Siemssen N, Laufs R. Parallel induction by glucose of adherence and a polysaccharide antigen specific for plastic-adherent *Staphylococcus epidermidis*: Evidence for functional relation to intercellular adhesion. Infect Immun. 1992;60(5):2048–57.

17. Vuong C, Gerke C, Somerville GA, Fischer ER, Otto M. Quorum‐Sensing Control of Biofilm Factors in *Staphylococcus epidermidis*. J Infect Dis. 2003;188(5):706–18.

18. Geiger T, Francois P, Liebeke M, Fraunholz M, Goerke C, Krismer B, et al. The Stringent Response of *Staphylococcus aureus* and Its Impact on Survival after Phagocytosis through the Induction of Intracellular PSMs Expression. PLoS Pathog. 2012;8(11):e1003016.

19. Brückner R. A series of shuttle vectors for *Bacillus subtilis* and *Escherichia coli*. Gene. 1992;122(1):187–92.

20. Pantůček R, Doškař J, Růžičková V, Kašpárek P, Oráčová E, Kvardová V, et al. Identification of bacteriophage types and their carriage in *Staphylococcus aureus*. Arch Virol. 2004;149(9):1689–703.

21. Rohde H, Burdelski C, Bartscht K, Hussain M, Buck F, Horstkotte MA, et al. Induction of *Staphylococcus epidermidis* biofilm formation via proteolytic processing of the accumulation-associated protein by staphylococcal and host proteases. Mol Microbiol. 2005;55(6):1883–95.
